# When man got his mtDNA deletions?

**DOI:** 10.1111/acel.12231

**Published:** 2014-06-04

**Authors:** Konstantin Popadin, Adeel Safdar, Yevgenya Kraytsberg, Konstantin Khrapko

**Affiliations:** 1 University of Geneva Medical SchoolGeneva, Switzerland; 2 Beth Israel Deaconess Medical Center, Harvard Medical SchoolBoston, MA, USA

## Abstract

Somatic mtDNA mutations and deletions in particular are known to clonally expand within cells, eventually reaching detrimental intracellular concentrations. The possibility that clonal expansion is a slow process taking a lifetime had prompted an idea that founder mutations of mutant clones that cause mitochondrial dysfunction in the aged tissue might have originated early in life. If, conversely, expansion was fast, founder mutations should predominantly originate later in life. This distinction is important: indeed, from which mutations should we protect ourselves – those of early development/childhood or those happening at old age? Recently, high-resolution data describing the distribution of mtDNA deletions have been obtained using a novel, highly efficient method (Taylor *et al*., 2014). These data have been interpreted as supporting predominantly early origin of founder mutations. Re-analysis of the data implies that the data actually better fit mostly late origin of founders, although more research is clearly needed to resolve the controversy.

mtDNA mutations, and in particular deletions, progressively increase with age and are suspected culprits of several age-related degenerative processes. Because there are hundreds or even thousands of mtDNA genomes per cell, increase in mutational load may include not only the increase in the number of cells containing mutant genomes, but also increase in the fraction of mutant mtDNA in each cell. Studies of mutational composition of individual cells showed that accumulation of mutations within a cell usually does not occur via accrual of random hits. Instead, mtDNA mutations ‘clonally expand’, that is, a single initial mutation multiplies within cell, replaces normal mtDNAs, eventually takes over the cell, and may impair its mitochondrial function. Expansion is possible because mtDNA molecules in a cell are persistently replicated, even in nondividing cells, where some of them are destroyed and replaced by replication of others. Half-life of murine mtDNA is on the order of several weeks (Korr *et al*., 1998). The result of clonal expansion is that different cells typically contain different types of mutations, while mutant genomes within a cell carry the same mutation. Mechanisms of expansion are still debated; possibilities range from neutral genetic drift to selection within the ‘population’ of intracellular mitochondria. In this commentary, we do not assume any particular mechanism and concentrate on the kinetics of expansion.

Because expansion takes time, it is possible that founder mutations of expanded mutant clones that compromise mitochondria at old age might have occurred early in life. Indeed, if expansion was a slow process taking about a lifetime to conclude (Fig. 1A, upper panel), then only those mutations that were generated early in life would have enough time to reach harmful intracellular concentration. In an utmost version of this scenario, there is little *de novo* mutagenesis and increase in mutations with age is mostly driven by clonal expansion of early founder mutations. The ‘slow’ scenario implies that, as far as mtDNA mutagenesis is concerned, we need to preserve mtDNA during early years or even during development and to be less worried about mutations that arise in older individuals.

**Figure 1:**
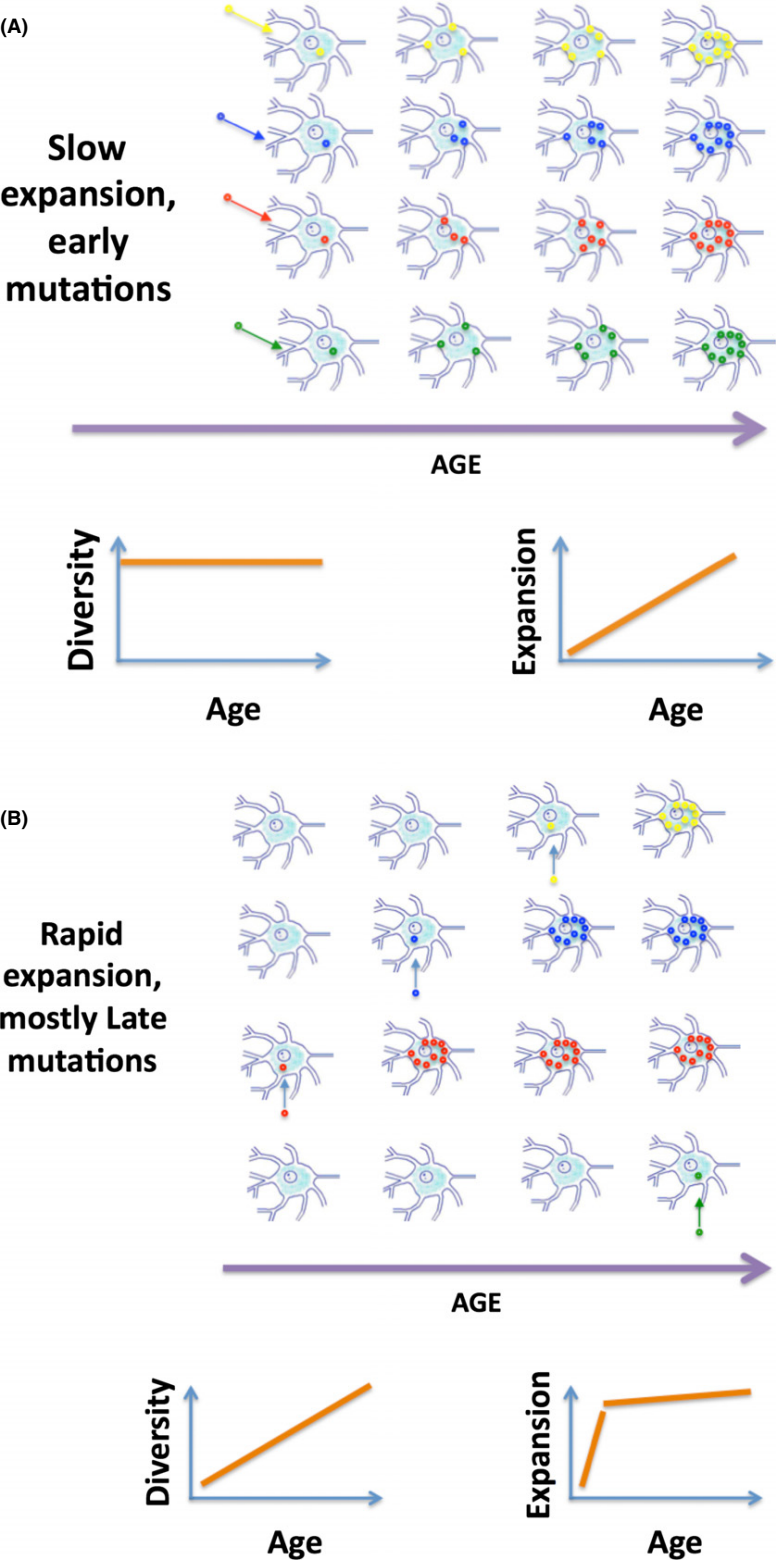
The ‘slow’ and the ‘fast’ expansion scenarios and the predicted changes in the diversity and extent of expansion of mtDNA mutations with age. Diversity and extent of expansion can be directly measured and used to distinguish between the two scenarios. mtDNA molecules with different deletions are depicted by small circles of different bright colors. Wild-type mtDNA molecules and cells that never get mutations are not shown for simplicity. Of course in a real tissue, mutant cells are surrounded by a great majority of nonmutant cells.

If, on the other hand, clonal expansion was rapid (Fig. 1B upper panel), then expanding mutations would swiftly fill up the cells in which founders had arisen and therefore stop expanding. Consequently, overall mutation load would soon ‘plateau off’ if mutants were not continuously occurring. So, in this scenario, the observed persistent increase in mutations with age must be driven by *de novo* mutagenesis, and most impairment in this scenario is caused by ‘late in life’ mutations, which therefore should be of primary concern. Despite importance of this question, there is still no consensus on which scenario is correct (Payne *et al*., 2011), (Khrapko, 2011), although the ‘early mutations’ hypothesis appeared more than a decade ago (Elson *et al*., 2001), (Khrapko *et al*., 2003).

Figure 1 schematically depicts two characteristics of the mutational dynamics that distinguish between the two scenarios. First, the *diversity,* that is, number of different *types* of deletions (Supplemental Note 0), remains constant in the slow scenario (Fig. 1A), but steadily increases in the fast scenario (Fig. 1B). Second, the extent of *expansion*, that is, the average number of mutant mtDNA molecules per clonal expansion, should increase steadily throughout the lifespan in the slow, early mutations scenario. In contrast, in the fast scenario, extent of expansion should increase rapidly early in life, up to the point when the earliest mutations had enough time to expand to the limits of their host cells and increase much slower thereafter.

Recent paper by Taylor *et al*. (Taylor *et al*., 2014) describes a new method called Digital Deletion Detection, based on enrichment of deletions by wild-type specific restriction digestion, massive single-molecule PCR in microdroplets, followed by next generation sequencing of the PCR products. This ‘3D’ approach for the first time provided a detailed frequency distribution for a large set of different deleted mtDNA molecules in human brain as a function of age. These data are sufficient to estimate diversity and level of expansion of mutations and therefore promise to help to distinguish between the fast and the slow scenarios. The authors found that a) the number of different types of deletions per sample (used as proxy of *diversity*) does not increase with age, while b) the ratio of total deletion frequency over the number of deletion types (used as proxy of *expansion*) does steadily increase with age. Consequently, the authors concluded that ‘diversity of unique deletions remains constant’ and that the ‘data supported the hypothesis that expansion of pre-existing mutations is the primary factor contributing to age-related accumulation of mtDNA deletions’, that is, the slow expansion scenario. We believe, however, that these data deserve more detailed analysis and more cautious interpretation.

A striking feature of the data (Taylor *et al*., 2014) is that the types of deletions found in any two samples are almost completely different (Supplemental Note 1). The same pattern has been previously observed in muscle (Nicholas *et al*., 2009). To explain this, consider that deletions originate mostly from individual cells each containing clonal expansion of deletion of a certain type. Because there are very many potential types of deletions and much fewer clonal expansions per sample, only a small proportion of possible types of deletions are found in each sample, which explains why two samples typically have almost no deletions in common. Similarly, any two cells with clonal expansions from the *same* sample usually carry different types of deletion. With this in mind, we will reconsider interpretation of the data.

First, consider *diversity* of deletions. Unfortunately, number of deletion types *per sample* normalized against the total number of deletions used by Taylor *et al*. as proxy of diversity is not an adequate measure. First, normalization against the total number of sampled deletion molecules is not justified because in a sample with clonal expansions, the number of types of deletions is not proportional to the number of sequenced molecules (Supplemental Note 2). Instead, the number of deletion types *per sample* is proportional to the size of the sample (i.e., the size of the tissue piece actually used for DNA isolation). Indeed, increasing sample size means including proportionally more cells with expansions. As discussed above, these additional cells contain different deletion types, so the number of deletion types will also increase roughly proportionally to the sample size. Sample size must be factored out of a rational measure of deletion diversity. The best proxy of sample size available in the original study (Taylor *et al*., 2014) is the number of mtDNA copies isolated from each sample. Thus, to factor out the sample size, we used the number of deletion types per 10^10^ mtDNA, (Fig. 2A). This corrected measure shows rather strong *(P < 0.0003)* increase in diversity of mtDNA deletions with age (Supplemental Note 3), which fits the ‘fast’ expansion scenario (Fig. 1B).

**Figure 2:**
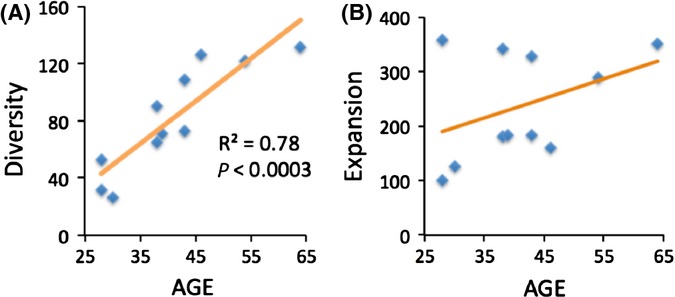
The observed changes in diversity and extent of expansion of mtDNA mutations in brain with age in Taylor *et al*. data. (A) Diversity of mtDNA deletions (number of deletion types per 10^10^ mtDNA) shows strong increase with age (*P* < 0.0003), corroborating the ‘fast’ expansion scenario (Fig. 1B). (B) The extent of expansion shows excessive variance and does not seem to support any of the two scenarios (neither ‘fast’ nor ‘slow’) to any significant extent. Interpretation of these data requires more detailed analysis.

Next, we revisited the extent of *expansion* of clonal mutations. As a measure of expansion, we used the average of the actual *numbers* of deleted molecules per deletion type, same as in Fig 1A,B. Note that this measure is different from ‘expansion index’ (Taylor *et al*., 2014), defined as deletion *frequency* per deletion type. This is essentially the same measure we use, additionally divided by the number of all mtDNA molecules in the sample. Unfortunately, ‘expansion index’ so defined systematically increases with decreased sample size. This is because deletion frequency is not expected to systematically increase with sample size, while the number of deletion types is, as shown in the previous paragraph. Thus, in particular because old samples in this set tend to be smaller (Supplemental Fig. 4A), this measure is biased.

Extent of expansion of mtDNA mutations is plotted versus age in Fig. 2B. Which theoretical expansion pattern, the ‘slow’ (Fig. 1A) or the ‘fast’ (Fig. 1B), better fits the actual data (Fig. 2B)? It looks like either fit is poor: the data are notoriously variable. We conclude that it is necessary to look beyond the coarse average measure of the expansion to interpret the data and explain the excessive variance (Supplemental Note 4).

The characteristic biphasic shape of the predicted ‘fast’ plot (Fig. 1B) results from the early large expanded mutations, which are absent in the ‘slow’ scenario (Fig. 1A). We therefore used the data (Taylor *et al*., 2014) to estimate the size of expansions (Supplemental Note 5, Supplementary Table S1) and in particular, to look for large expansions in young tissue. Indeed, young samples do contain large clonal expansions, and there are four expansions more than 1000 copies in samples 30 years and younger (Table S1). This is consistent with our own observations of large expansions of deletions in single neurons of the young brain using a different approach – single-molecule amplification (Kraytsberg *et al*., 2006). In other words, although rapid expansion pattern in Fig. 2B is obscured by large variance of the data, the hallmarks of fast expansion, that is, large early mutant expansions, are present in the tissue.

An aspect of the data, however, is at odds either with the fast or the slow scenario. The distribution of expansion sizes at any age is rather gradual; that is, there is a large proportion of expansions of intermediate sizes, ranging from smallest detectable (typically about 10 molecules) up to those more than 1000 molecules. In contrast, according to the ‘slow’ expansion scenario, all expansions should be of approximately the same size, which should increase with age, turning ‘large’ at approximately the same time. The fast scenario, also in contradiction with observations, predicts that proportion of mutants contained in expansions of intermediate sizes markedly decreases with age (Supplemental Note 6).

If neither of the scenarios fits the data, what kind of mutational dynamics could be responsible for the observed distribution (Taylor *et al*., 2014)? We believe that most plausible is a ‘mixed’ scenario, where expansions are fast in some cells and slow in other (‘fast’ and ‘slow expanders’, correspondingly), probably with the whole spectrum of expansion rates in between. Expansion rates may differ between cell types or between cells of the same type differing in individual activity, stress, levels of ROS, length of deletion (Fukui & Moraes, 2009), etc. An example of such a difference is given by myoblasts, which, unlike their descendant myofibers, support only very slow, if any, expansion of mtDNA deletions (Moraes *et al*., 1989).

What does this mean with respect to the question in the title of this commentary – when do mtDNA deletions arise? In fact, if we accept the mixed scenario, then it follows that the share of late mutations is at least significant. Indeed, if late mutations played little role, then accumulation of mutations should have been markedly decelerating with age. This is because ‘fast expander’ cells are saturated with mutations early in life and increase in mutation load at older age is driven by progressively ‘slower expanders’, meaning slower increase in mutational load. In contrast with this prediction, accumulation of deletions observed in most tissues appears to aggressively accelerate with age and is traditionally approximated with an exponent. This is also true for the Taylor *et al*. (2014), which are better fit by accelerating curves than they are by linear function (Fig. S6). The fraction of deleted mtDNA increases over the lifespan by up to four orders of magnitude in highly affected brain areas such as substantia nigra and about three in less affected, such as cortex (Meissner *et al*., 2008). In principle, even such a dramatic increase in mutant fraction might be entirely driven by expansion of early founder mutations in slow scenario. Neurons contain thousands of mtDNA copies, so expansion alone could potentially sustain about four orders of magnitude increase in mutant fraction from single founder mutants mtDNA to fully mutant cells. However, *accelerated* accumulation of (expanded) mutations in mixed scenario can only be explained by generation of *de novo* mutations at older ages.

The reality is probably more complicated than idealized scenarios considered above. For example, cells with expanded mutations may die preferentially. If true, this would make fast scenario/late origin even more plausible. Indeed, that would mean that the actual number of mutations that have reached full expansion at any age is higher than observed (extra mutations being those that had died), implying that mutations expand faster than it appears. Other refinements of the model are certainly possible. However, notable variability of the data makes testing hypotheses, in particular, complex ones, difficult. Excessive variability of data on mtDNA deletions has been observed before, for example Meissner *et al*., 2008, but have never been duly explored. Lack of replicate analyses hampers understanding of the source of variance and of the shape of the frequency distributions of mutations. The latter are indispensable for interpreting the data. Future studies seeking to explain dynamics of mutations with age must include multiple replicate measurements (Supplemental Note 7).

In conclusion, re-analysis of the data (Taylor *et al*., 2014) challenges the authors’ inference that diversity of unique deletions remains constant with age and that expansion of pre-existing deletions is the primary factor contributing to age-related accumulation of mtDNA deletions. The data are more consistent with increasing diversity of deletions and significant impact of mutagenesis at older age. However, the issue is far from being solved, in part because of high variability of the data, and it awaits more detailed studies (Supplemental Note 7).

## Supporting Information

**Fig. S1** Predicted change in the number of different deletion types discovered in a sample as a function of the number of deletion molecules analyzed, depending on the shape of the assumed frequency distribution of deletions. **Fig. S2** Fraction of deletion types represented by two or more molecules is a rough measure of ‘saturation’ of the coverage of the deletion repertoire by deletions sequenced for a given sample. **Fig. S3** Apparent proportionality between number of deletion types discovered per sample and the number of deleted molecules sequenced per sample is confounded by the sample size (i.e., per 1010 mtDNA copies). **Fig. S4** A. Number of mtDNA copies per sample is negatively correlated with age among the subset of samples subjected to ddPCR. **Fig. S5** Fraction of mDNA deletions contained in intermediate sized (<500 copies) clonal expansions as a function of age. **Fig. S6** Increase in mutant fraction of deletions accelerates with age.

**Table S1** Estimations of absolute sizes of clonal expansions.
