# Supplementary material for: When man got his mtDNA deletions?
Source: Aging Cell. 2014 Jun 4;13(4):579–82. doi: 10.1111/acel.12231 (PMC4326951; doi:10.1111/acel.12231)
Supplement: Supplementary file 1 — Fig. S1 Predicted change in the number of different deletion types discovered in a sample as a function of the number of deletion molecules analyzed, depending on the shape of the assumed frequency distribution of deletions. Fig. S2 Fraction of deletion types represented by two or more molecules is a rough measure of ‘saturation’ of the coverage of the deletion repertoire by deletions sequenced for a given sample. Fig. S3 Apparent proportionality between number of deletion types discovered per sample and the number of deleted molecules sequenced per sample is confounded by the sample size (i.e., per 1010 mtDNA copies). Fig. S4 A. Number of mtDNA copies per sample is negatively correlated with age among the subset of samples subjected to ddPCR. Fig. S5 Fraction of mDNA deletions contained in intermediate sized (<500 copies) clonal expansions as a function of age. Fig. S6 Increase in mutant fraction of deletions accelerates with age. [file acel0013-0579-sd1.pdf]

When man got his mtDNA deletions?

On-Line Supplement

K. Popadin[1], A. Safdar[2], Y. Kraytsberg[2], K. Khrapko[2]*.

[1]University of Geneva Medical School
[2]Beth Israel Deaconess Medical Center, Harvard Medical School
* Corresponding author: kkhrapko@gmail.com

Supplementary Note 0: The term "Type of deletion" implies the exact identity of the deletion molecule, as defined by the exact sequence of its breakpoints, i.e. sites where the deleted fragment was cut out of mtDNA. Note that Taylor et. al., (Taylor et al., 2014) uses the term "unique deletion" to describe what we prefer to call "deletion type".

Supplementary Note 1: Note that this is not true for deletions that are created at high mutational rates, e.g. the "common deletion", because such deletions do happen independently in many cells of a sample. This makes common deletion non-informative, and we excluded the "common deletion" part of the data (Taylor et al., 2014) from consideration.

Supplementary Note 2. Normalization of diversity by the number of sequenced molecules is not justified.
For the measure of diversity used by Taylor et al. in their Figure 5B, the number of deletion types discovered in a sample (which is the measure used by us), was additionally *divided by the number of deletion molecules from that sample that were sequenced* (i.e., which were subjected to 3D analysis, which was about 1% of all deletion molecules in a sample). The reason for this additional normalization was an assumption that the number of deletion *types* discovered in a sample is proportional to the number of deleted molecules sequenced from that sample.

Whether this assumption is correct or not, depends, however, on the frequency distribution of the types of deletions. For example, if the number of deletion types is much larger that the number of sequenced molecules and types are represented by similar numbers of molecules, then, indeed, the more molecules are sequenced the more deletion types are discovered (**Fig. S1a**), because each type of deletion in this case is typically represented by a single molecule.

But the same assumption is obviously not correct if the number of deletion types in a sample is low compared to the number of deletion molecules sequenced. In this case, deletions of the same type will be sequenced over and over again and the curve will reach a plateau (**Fig. S1b**). A more realistic distribution is a combination of the two described above: a relatively small subset of high frequency ("expanded") deletions plus a large number of low frequency ("non-expanded") deletions. This kind of distribution results in a mixed curve of the type shown in **Figure S1c**. We have observed this phenomenon as we were sequencing progressively more deleted molecules from muscle samples (Nicholas et al., 2009), so we believe that distribution of the **Figure S1c** type is most likely to represent the real tissue. Normalizing by the number of sequenced molecules in this situation would create a strong bias: "diversity" would appear to decrease with the number of sequenced molecules.

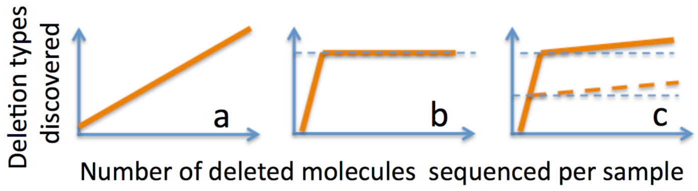

Figure S1*. Predicted change of the number of different deletion types discovered in a sample as a function of the number of deletion molecules analyzed, depending on the shape of the assumed frequency distribution of deletions. Details in the text (****Supplementary Note 2****) a) The number of deletion types is much larger that the number of sequenced deleted molecules and types are represented by similar numbers of molecules. b) The number of deletions types is low compared to the number of deletions sequenced. c) a relatively small subset of high frequency ("expanded") deletions plus a large number of low frequency ("non-expanded") deletions. Broken orange line pertains to a sample half the size*.

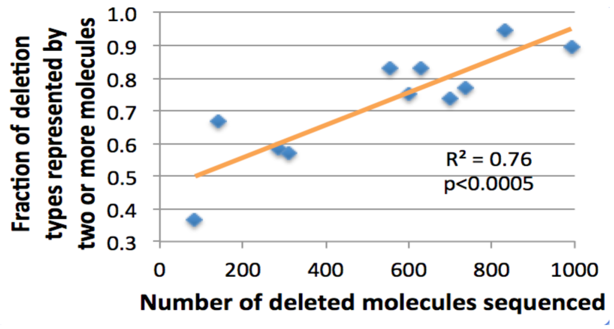

Figure S2*. Fraction of deletion types represented by two or more molecules is a rough measure of "saturation" of the coverage of the deletion repertoire by deletions sequenced/sequenced for a given sample. (See text)*

So, to normalize or not to normalize by depends on the frequency distribution of the deletion types. In Taylor et. al data, a great majority of deletion types are represented by a fair number of independent deleted molecules (**Supplementary Table**). On average over 70% of deletion types are represented by 2 or more molecules. Furthermore, percentage of deletion types represented by two or more molecule strongly increases with the number of sequenced molecules (**Fig S2**), which hints that coverage level is such that most deletion types that have been sequenced once are about to be sequenced twice as more deletions are sequenced. Increase of the number of sequenced molecules in this situation will only weakly

affect the number of deletion types. Therefore normalization by the number of sequenced molecules is not justified. Moreover, the use of such normalization is expected to create a bias. We therefore avoided using normalization by the number of sequenced deletions in presenting the Taylor et. al data in our Figure 3A.

In apparent contradiction with the conclusion that number of deletion types discovered in a sample should only marginally increase with the number of sequenced deleted molecules, the two variables appear strongly (p~0.02) correlated (**Fig. S3a**). The caveat, however, is that **Fig. S3a** does not pertain to a single sample as **Fig. S1a** does, but relates to different samples, apparently *with different frequency distributions of deletions*. Note that, as discussed in the main text, such distributions are affected by the size of the sample. Smaller sample means fewer expansions, which means that number of deletion types plateaus at a proportionally lower level. A curve expected for a sample half the size is sketched by a broken line in **Fig. S1c**. Samples of different sizes, thus can only be compared upon normalization the number of deletion types by the size of the sample. Once this is done, proportionality between the number of sequenced deletions and the deletions types essentially ceases to exist (**Fig. S3b**).

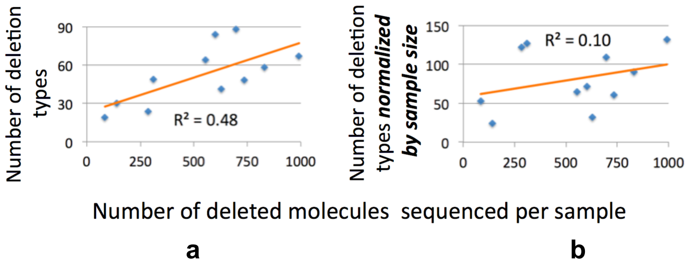

Fig S3. *Apparent proportionality between number of deletion types discovered per sample and the number of deleted molecules sequenced per sample is confounded by the sample size (i.e. per 10^{10} mtDNA copies)*.

Supplementary Note 3:
The change of the correlation between diversity and age from insignificant negative in Taylor et al. (Fig. 5B), to highly significant positive in **Fig 2A** depends on two factors. One is that we *in*cluded normalization of diversity measure by the sample size and second – that we *ex*cluded normalization by the number of deletions sequenced.

Interestingly, total mtDNA copy number appears to negatively correlate with age (**Figure S4A**). Apparently, this correlation is purely accidental: it just happened so that old samples used for sequencing analysis were on average smaller than young ones, while in general old samples are not smaller than young (**Fig. S4B**). This correlation does imply a bias that needs to be corrected.

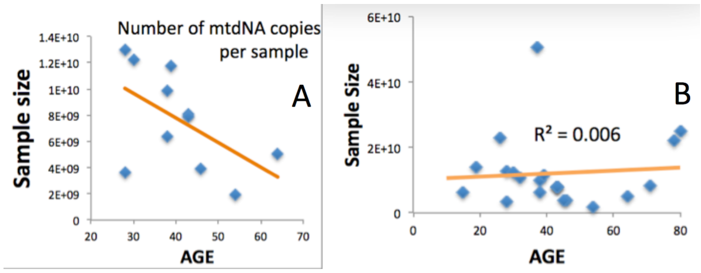

Figure S4. *A. Number of mtDNA copies per sample is negatively correlated with age among the subset of samples subjected to ddPCR. This is not the case for samples in general –* ***Fig S4B*** *below shows all samples. The correlation shown in* ***Fig S4A*** *is obviously very weak, however, it imposes (together with normalization bias –* ***Supplementary Note 2****) strong enough influence to convert non-significant negative correlation of diversity vs. age shown in Fig5B (Taylor et al., 2013) into highly significant positive correlation of* ***Fig2A*** *in this commentary. B. There is no overall correlation between mtDNA per sample and age overall.*

Supplementary Note 4. Excessive variance.
The excessive variance of the extent of expansion may be at least in part be explained by sampling error: indeed, there are very few large expansions per sample, especially in the young. The issue of excessive variance of the data and its sources are further discussed in **Supplementary Note 7.1**.

Supplementary Note 5 (Comments to the Supplementary Table S1): Estimating absolute sizes of clonal expansions.

Supplementary Table S1 (located in a separate attachment) contains processed data (Taylor et al., 2014). We processed the data to reveal the absolute sizes of clonal expansions. To do this, we multiplied partial mutant fraction of each deletion in a sample by the total number of mtDNA copies in that sample. Partial mutant fractions were calculated by multiplying overall mutant fraction of a sample by the fraction of *reads* corresponding to a deletion in question among all deletion reads. **Supplementary Table S1** also contains sample identification with age, number of mtDNA copies and overall mutant fraction in convenient format.

Justification for this way of estimation is that deletions originate from individual cells each containing clonal expansion of deletion of a certain type . Because there are much more potential types of deletions than clonal expansions per sample, only a small proportion of possible types of deletions are found in each sample. As a result, two cells from the *same* sample that carry expanded deletions rarely carry the same type of deletion. If so, all deletion molecules of the same type present in a sample most likely originate from the same clonal expansion. This

means that partial mutant fraction of a given deletion multiplied by the total number of mtDNA copies in a sample gives the size of the corresponding clonal expansion.

It should be noted that the number of reads representing a certain deletion, depends, in addition to the actual partial mutant fraction of the deletion in the sample, on the relative amplification efficiency of the corresponding PCR fragments, and may result in considerable differences between deleted molecules (Jason Bielas and Sean Taylor, personal communication). These effects should result in an additional variance superimposed on whatever variance is already present in the data for other reasons. It is therefore particularly reassuring that we are able to see clear age-dependent trends like those shown in **fig 2A**, as these trends are apparent *despite* this additional source of variance.

Supplementary Note 6. Expected size distribution of clonal Expansions.
The "fast" scenario predicts that proportion of mutants contained in expansions of intermediate sizes should markedly decrease with age, while majority of mutant molecules at old age should be found in cells with large fully grown expansions. For example, if mutant typically fully expands in about 10 years, and mutations were occurring at a constant rate, then, in a 10-year old, essentially all expansions would be of intermediate size, from completely non-expanded mutations that newly occurred at age 10 to almost completely expanded that occurred at year 0. In a 60-year old individual, about 5/6 of mutants (ones that occurred by year 50) will be fully expanded, while 1/6 (occurred from 50 to 60 years) will be still undergoing expansion and thus represented by expansions of intermediate sizes. The faster expansion rate, the lower the expected proportion of intermediate mutations and the faster its decrease with age. However, in contradiction with both the slow and the fast scenario predictions, intermediate sized expansions at any age typically contain a majority of mutations, and their proportion barely changes with age (**fig. S5**).

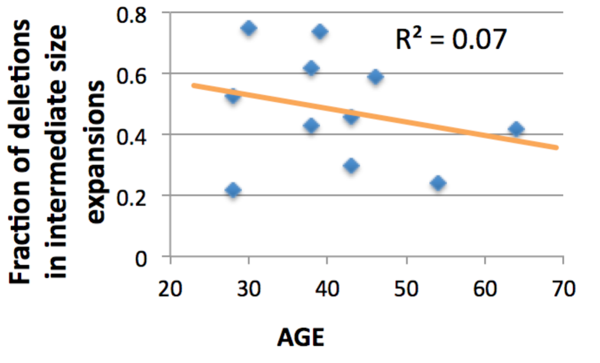

Figure S5. *Fraction of mDNA deletions contained in intermediate sized (<500 copies) clonal expansions as a function of age*.

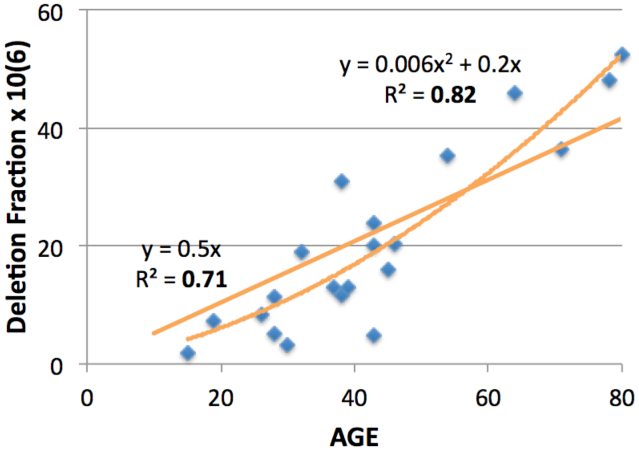

Figure S6. *Increase of mutant fraction of deletions accelerates with age. The full data* (Taylor et al., 2013) *including non-sequenced samples has been included in this plot. Of note, overall increase of deletions and the extent of acceleration is lower than typical values reported, e.g. (Meissner et al., 2008). This may be related to the fact that Taylor et al. data do not include very young individuals, which typically present with extremely low deletion fractions*.

Supplementary Note 7. *Future prospects: Replicate experiments needed*.

Perhaps the most important conclusion from our re-analysis of the data (Taylor et al., 2014) is that there is great need for replicate measurements. Interpretation of its otherwise excellent data was hampered because most measurements have been done only once. Replicate experiments will help to:

1. Resolve excessive variance of the data.

Large variation in mutant fraction, diversity, and expansion levels between samples of similar age are observed in (Taylor et al., 2014) and other studies (e.g. (Meissner et al., 2008)). This large variance makes many correlations less convincing, difficult to interpret (e.g. Fig 2B) and perhaps is masking certain important correlations altogether. Does this variance result from real differences between individuals of the same age? Alternatively the variation may be random, such as random experimental error, or local differences of deletion levels within tissue (i.e. a local high-mutation area within tissue happened to be sampled in one individual and low mutation area in the other, though overall mutational levels in the two individuals are similar). Finally, this can be the "sampling error", i.e. variation related to a low number of large clonal expansions per sample. For example, there are 3 expansions over 1000 mutations in patient 1 (28 yo), mutant fraction 10^{-6} and none in patient 2, also 28 yo, so perhaps not surprisingly, mutant fraction is lower: $5x10^{-7}$ (**Table S1**). Inclusion of more replicates will reduce random variance. If, in contrast, multiple sampling reveals

reproducible significant differences between individuals not related to age, that would be a result worth a follow up.

2. Test assumptions regarding distributions of mutations.

a. Key to the logic of this commentary was that the number of deletion types is proportional to the sample size. To prove that, we used complicated indirect reasoning (text related to Figure 2A). Instead we could get a clear direct answer if tissues were sampled two or more times. Simply put, if pooling deletion types from two replicates (which, taken together, imitate a sample twice the size of each of replicates) results in doubling of deletion types, then our conclusion that diversity of deletion types needs to be normalized by sample size is correct. Otherwise, if deletion types in two replicates are highly redundant and, as a result, the number of deletion types in pooled replicates is much lower than twice the number in each of them, then this assumption is incorrect. Certainly intermediate situations are possible and the answer may be different in different individuals, and even in different brain areas of the same individual.

b. Another important assumption used in our argument was that increase in the number of deletion molecules sequenced is not an independent correlate of the number of deletion types and thus diversity should not be normalized by this number (**Supplementary Note 2**). Instead of making complicated indirect assumptions regarding the distribution of deletion types, replicate measurements (if were available) could have been used to get the answer. By reasoning similar to that used in the previous paragraph, if the number of deletion types in two pooled replicates is approximately twice of that in each of them, then assumption is incorrect and normalization is needed. Conversely, if deletion types in two replicates are highly degenerate, do not "add up", and the number of deletion types in pooled sample are about the same as in each of them, then assumption is correct and normalization is not needed.

The replicate experiments needed are of two types: the "biological" and the "instrumental". The "biological" replicates are measurements (including replicate 3D determination of the breakpoint distribution) from DNA isolated from different tissue samples from the same individual, e.g. cuts from different sides of the same tissue piece. We use the word "biological" replicates, because they estimate natural biological variance of mutational load and diversity. Biological replicates permit to determine whether variance is random or reproducible (**Supplementary Note 7.1**) and whether the number of deletion types is proportional to the sample size (**Supplementary Note 7.2.a**).

"Instrumental" replicates, that is, procedure repeated independently using two aliquots of DNA taken from the same DNA isolate. These reveal any variance created by the technique and are very desirable in studies involvng a new method. "Instrumental" replicates also permit to determine whether increase in the number of deletion molecules sequenced is not an independent correlate of the number of deletion types (**Supplementary Note 7.2.b**)
